# Accuracy of imputation using the most common sires as reference population in layer chickens

**DOI:** 10.1186/s12863-015-0253-5

**Published:** 2015-08-18

**Authors:** Marzieh Heidaritabar, Mario P. L. Calus, Addie Vereijken, Martien A. M. Groenen, John W. M. Bastiaansen

**Affiliations:** Animal Breeding and Genomics Centre, Wageningen University, P.O. Box 338, 6700 AH Wageningen, the Netherlands; Animal Breeding and Genomics Centre, Wageningen UR Livestock Research, P.O. Box 338, 6700 AH Wageningen, the Netherlands; Hendrix Genetics Research, Technology and Services B.V., P.O. Box 114, 5830 AC Boxmeer, the Netherlands

**Keywords:** Imputation accuracy, Layer chickens, Reference population design

## Abstract

**Background:**

Genotype imputation has become a standard practice in modern genetic research to increase genome coverage and improve the accuracy of genomic selection (GS) and genome-wide association studies (GWAS). We assessed accuracies of imputing 60K genotype data from lower density single nucleotide polymorphism (SNP) panels using a small set of the most common sires in a population of 2140 white layer chickens. Several factors affecting imputation accuracy were investigated, including the size of the reference population, the level of the relationship between the reference and validation populations, and minor allele frequency (MAF) of the SNP being imputed.

**Results:**

The accuracy of imputation was assessed with different scenarios using 22 and 62 carefully selected reference animals (Ref_22_ and Ref_62_). Animal-specific imputation accuracy corrected for gene content was moderate on average (~ 0.80) in most scenarios and low in the 3K to 60K scenario. Maximum average accuracies were 0.90 and 0.93 for the most favourable scenario for Ref_22_ and Ref_62_ respectively, when SNPs were masked independent of their MAF. SNPs with low MAF were more difficult to impute, and the larger reference population considerably improved the imputation accuracy for these rare SNPs. When Ref_22_ was used for imputation, the average imputation accuracy decreased by 0.04 when validation population was two instead of one generation away from the reference and increased again by 0.05 when validation was three generations away. Selecting the reference animals from the most common sires, compared with random animals from the population, considerably improved imputation accuracy for low MAF SNPs, but gave only limited improvement for other MAF classes. The allelic R^2^ measure from Beagle software was found to be a good predictor of imputation reliability (correlation ~ 0.8) when the density of validation panel was very low (3K) and the MAF of the SNP and the size of the reference population were not extremely small.

**Conclusions:**

Even with a very small number of animals in the reference population, reasonable accuracy of imputation can be achieved. Selecting a set of the most common sires, rather than selecting random animals for the reference population, improves the imputation accuracy of rare alleles, which may be a benefit when imputing with whole genome re-sequencing data.

**Electronic supplementary material:**

The online version of this article (doi:10.1186/s12863-015-0253-5) contains supplementary material, which is available to authorized users.

## Background

Using dense single nucleotide polymorphism (SNP) panels, genomic selection (GS) and genome-wide association studies (GWAS) have become common in animal and plant genomic breeding programs. Both GS and GWAS exploit linkage disequilibrium (LD) between SNPs and causative mutations. Increasing the density of SNP panels is therefore expected to contribute to improved accuracies of genomic prediction and GWAS [1, 2]. However, higher density of SNPs means higher genotyping cost which is still a key constraint in implementing GWAS and GS in animal breeding programs. To overcome this constraint, selection candidates can be genotyped for a low-density SNP panel after which a higher density SNP panel is obtained through imputation.

Animals may be genotyped for different SNP chips due to the expansion of available genotyping technologies, for design reasons, or due to the coexistence of several genotyping products [3]. Thus far, different SNP chips have been developed for chicken. For instance, the publicly available chicken 60K SNP chip [4] from Illumina and the 600K SNP chip [5] from Affymetrix. Another SNP chip, containing 42K SNPs, has been developed as a proprietary tool in chickens [6]. These SNP chips have been widely used for purposes such as GWAS [7, 8], GS [9–13], fine mapping of quantitative trait loci (QTL) [14] and identification of selection signals [15]. Because of genetic variation within and between domesticated and commercial chicken breeds [16] and because of differences in LD patterns between different chicken breeds [17], a higher density SNP chip would be useful to address different purposes mentioned above (GS, GWAS, identification of selection signals and fine mapping of QTL) in a diverse range of chicken breeds and populations. In the future, additional SNP chips or even whole-genome sequence data may replace the current SNP chip data in avian genetic and genomic studies. As higher density SNP chips are put into use, the re-genotyping of previously genotyped individuals with these new chips would be costly. Imputation from the lower density chip towards the higher density chip could then be a cost-effective strategy. With two different SNP chips, a combined dataset with all SNPs genotyped on all individuals would be desired. Imputation could be used, but the feasibility and accuracy of SNP imputation between the SNP chips needs to be tested. Druet et al. [3] performed imputation between two SNP chips in cattle data, where the SNPs specific to the Illumina Bovine SNP50 (50K) chip were imputed for Dutch Holstein bulls that were genotyped using a custom-made 60K Illumina chip (CRV, Arnhem, the Netherlands) and vice versa [3]. Their results showed an imputation accuracy of 99 %. Imputation accuracy is of special interest for SNPs that have low minor allele frequency (MAF). Many studies that used SNP chip data [18–23] and also sequence data [24] to perform imputation have demonstrated lower imputation accuracy for SNPs with low MAF. However, the effect of reference population design on imputation accuracy of low MAF SNPs is largely unknown. Using simulation, Meuwissen and Goddard [25] found that the error rate was much improved when relatives were sequenced, and Khatkar et al. [26] suggested that selecting animals for genotyping based on pedigree is a strategically optimised method if pedigree information is available.

Several factors influence the accuracy of imputation including the genetic relationship between the animals in the reference and validation populations [27], the size of reference population [27], MAF of the SNP to be imputed [18], the proportion of missing genotypes on the low and high-density panel [28], the population structure and levels of LD [29], the imputation method and, if applicable, the parameter settings of the applied imputation algorithm [30]. One important factor is the genetic relationship between the animals in the reference and validation populations [27, 31]. When close relatives of target animals are genotyped at high density, the missing SNPs can be recovered through linkage and segregation analysis [32], where haplotypes can be traced across generations of directly related individuals by the Mendelian inheritance rules. The algorithms used for imputation use either LD information such as Beagle [33] and IMPUTE2 [34] or both LD and pedigree information such as AlphaImpute [35]. If a pedigree-free imputation method is used, the most important factors to increase the accuracy of imputation are: the size of the reference population and the availability of a representative reference population which maximises the accuracy of imputation and captures the highest proportion of genetic variation in the validation population.

Few studies have investigated imputation accuracy in poultry compared with other livestock species (see review [36]). Thus far, they have demonstrated that the application of imputation methods is effective in chickens. Comparing imputation accuracies across studies is difficult, since applied imputation softwares, size of reference populations, imputation measures, density panels, and population-specific parameters (e*.*g*.* LD and effective population size (*N*_*e*_)) differ substantially across studies. In general, high imputation accuracies were found in broiler chickens (ranging from 0.94 to 0.99) [37, 38] and also in brown egg layer chickens (ranging from 0.68-0.97) [39–41]. Most studies in chicken imputed missing genotypes from a very low density such as 384, 1K or 3K to a medium-density (20K, 36K or 60K). For instance, Wang et al. [38] and Hickey et al. [37] imputed from 384 SNPs to 20K and 36K, respectively. Vereijken et al. [39] imputed from three low-density panels (384, 1K and 3K) to 57K on six chromsomes of brown layer chickens. This study had two objectives. The first was to investigate the accuracy of imputation of 60K genotypes from lower density SNP panels (3K and 48K) using a small reference population of the most common sires. Imputation from 48K to 60K was performed not only to assess the impact of having a higher density panel as reference (compared with 3K) on imputation accuracy, but also to mimic the imputation of genotypes between two different SNP chips with similar densities. The second was to investigate the factors that affect imputation accuracy, namely: the size of reference population, the level of genetic relationship between the reference and validation populations, and the MAF of imputed SNP.

## Results

**Fig. 1:**
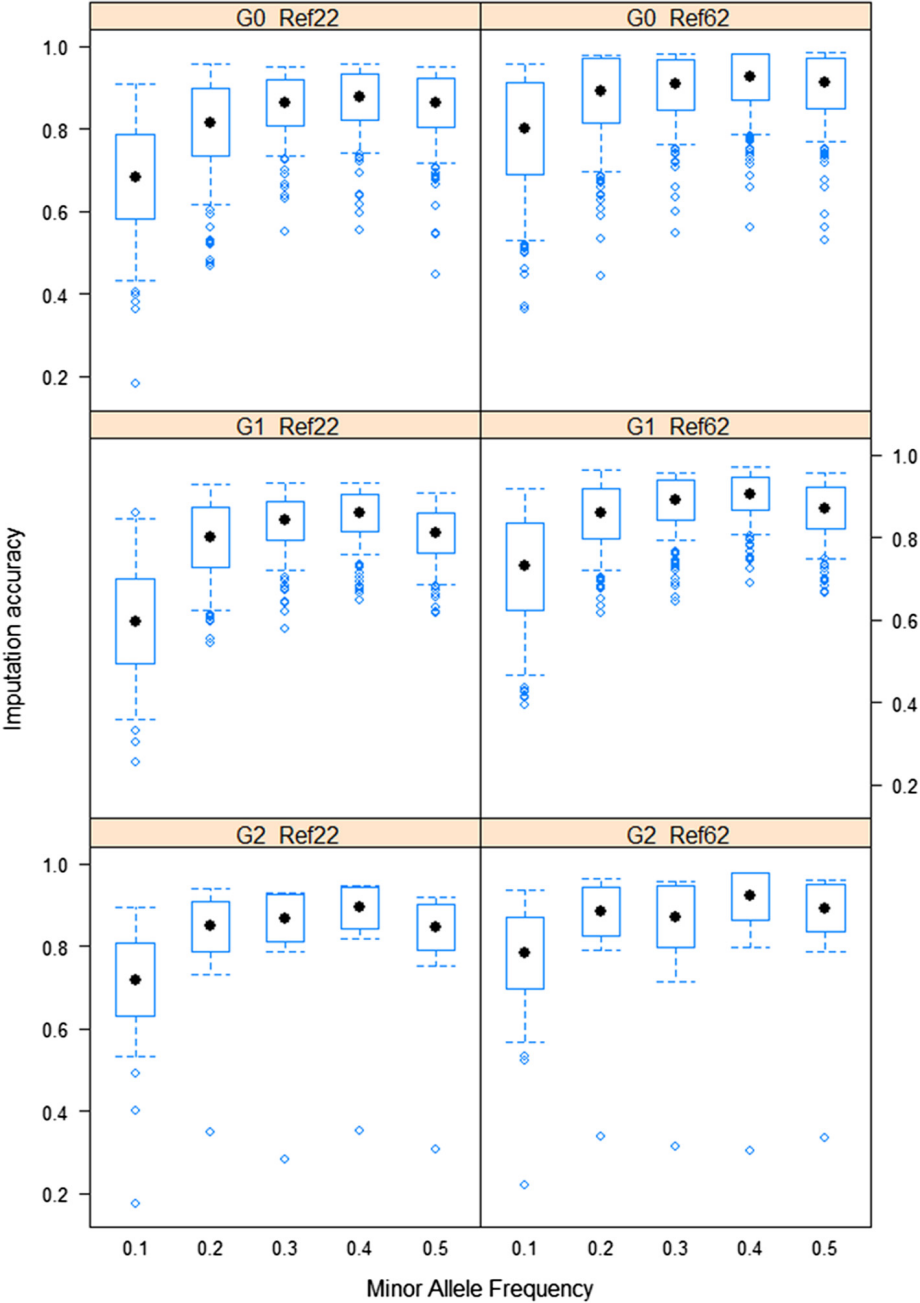
Imputation accuracies in G0, G1 and G2 for 48K to 60K scenario. Imputation accuracies (r_corrected_) for different MAF classes and different reference sizes for G0, G1 and G2 validation populations. The x-axis represents different classes of MAF and y-axis shows the imputation accuracies. The black dots are the mean imputation accuracies across individuals in each MAF class

In this study, the accuracy of imputation to 60K genotypes from lower density SNP panels (3K and 48K) was assessed in genotype data from GGA1 of layer chickens, when using a small reference population of the most common sires that are influential in the validation population. In addition, we evaluated the factors affecting imputation accuracy such as the size of reference population, the level of genetic relationship between the reference and validation populations (imputation in three discrete generations), and the MAF of imputed SNPs. Animal-specific imputation accuracy (r_corrected_) was used as the measure of imputation accuracy. For the 3K to 60K scenario, imputation accuracy ranged from 0.46 to 0.63 (Table 1). For the 48K to 60K scenario, imputation accuracies in the first generation of the validation population (G0) ranged from 0.68 for MAF class < 0.10 to 0.88 for MAF class 0.3-0.4 with only 22 animals (Ref_22_) in the reference population (Table 2, Fig. 1). Increasing the reference population size to 62 animals (Ref_62_) improved the accuracies to values from 0.80 to 0.93 for the same range of MAF classes (Table 2, Fig. 1). From G0 to G1, imputation accuracies decreased to 0.60 for MAF class < 0.10 and to 0.86 for MAF class 0.3-0.4 when Ref_22_ was used (Table 2, Fig. 1). From G1 to G2, imputation accuracies increased to 0.72 for MAF class < 0.10 and to 0.89 for MAF class 0.3-0.4 when Ref_22_ was used (Table 2, Fig. 1). Similar to the results for G0, imputation accuracies substantially increased for G1 and G2 by increasing the size of reference population in these generations (Table 2, Fig. 1).

**Table 1:** Animal-specific imputation accuracy (r_corrected_) on GGA1 for 3K to 60K scenario

Validation population	Ref_22_	Ref_62_
G0^1^	0.50	0.63
G1^2^	0.46	0.58
G2^3^	0.50	0.60

^1^ First generation of genomic selection experiment

^2^ Offspring of G0

^3^ Offspring of G1

**Table 2:** Animal-specific imputation accuracy (r_corrected_) and the standard errors on GGA1 for different MAF classes in G0, G1 and G2 validation populations (48K to 60K scenario)

Validation population		
G0^1^		
MAF^2^ class	Ref_22_	Ref_62_
0.008-0.1	0.68 (0.005)^a^	0.80 (0.006)
0.1-0.2	0.82 (0.004)	0.89 (0.004)
0.2-0.3	0.86 (0.003)	0.91 (0.003)
0.3-0.4	0.88 (0.003)	0.93 (0.003)
0.4-0.5	0.86 (0.003)	0.91 (0.003)
G1^3^		
MAF class	Ref_22_	Ref_62_
0.008-0.1	0.60 (0.005)	0.73 (0.005)
0.1-0.2	0.80 (0.004)	0.86 (0.003)
0.2-0.3	0.84 (0.002)	0.89 (0.002)
0.3-0.4	0.86 (0.002)	0.91 (0.002)
0.4-0.5	0.81 (0.003)	0.87 (0.002)
G2^4^		
MAF class	Ref_22_	Ref_62_
0.008-0.1	0.72 (0.007)	0.78 (0.007)
0.1-0.2	0.85 (0.005)	0.88 (0.005)
0.2-0.3	0.87 (0.005)	0.87 (0.006)
0.3-0.4	0.89 (0.004)	0.92 (0.005)
0.4-0.5	0.85 (0.005)	0.90 (0.005)

^1^ First generation of genomic selection experiment

^2^ Minor allele frequency

^3^ Offspring of G0

^4^ Offspring of G1

^a^ The values in parentheses are standard errors

### Imputation from 3K to 60K

Imputation based on a lower density SNP panel in the validation population, from 3K instead of 48K, resulted in lower imputation accuracies, as expected (Table 1). In comparison with the 48K to 60K scenarios (Table 2, Table 5), the 3K to 60K scenario gained more in imputation accuracies from enlarging the reference population (Table 1). The increase in imputation accuracies from Ref_22_ to Ref_62_ was 0.13 (0.50 to 0.63), 0.12 (0.46 to 0.58) and 0.10 (0.50 to 0.60) for G0, G1 and G2 (Table 1), respectively.

### Factors affecting the imputation accuracy

#### Size of reference population

As expected, accuracy of imputation increased as the size of the reference population increased. The increase in average imputation accuracies (average across MAF classes) from Ref_22_ to Ref_62_ was 0.07 (0.82 to 0.89), 0.07 (0.78 to 0.85), and 0.04 (0.83 to 0.87) for G0, G1 and G2, respectively (Table 2, Fig. 1).

#### Selection of animals for the reference population

Animals for Ref_22_ were selected for being influential, having the highest relationships with animals in the validation population. The proportion of diversity represented by the 62 sires and maternal grandsires of G0 are in Additional file 1: Table S2. The 22 and 62 sires and maternal grandsires captured 39.85 % and 75.54 % of genetic variation in the target population. In comparison, a subset of 22 randomly selected animals captured between 0.68 % and 3.36 % (on average 2.10 % across 10 subsets) of the genetic variation in the target population. The biggest impact from randomly selecting 22 animals in the reference was observed for MAF class < 0.10, where accuracy dropped by 0.07 (Table 3). A drop of 0.03 was observed for MAF class 0.4-0.5. The other MAF classes showed no changes in accuracy.

**Table 3:** Animal-specific imputation accuracy (r_corrected_) with 22 randomly selected animals (Ref_22rand_) in the reference population

MAF^1^ class	Ref_22rand_^a^
0.008–0.1	0.61 (0.006)^b^
0.1–0.2	0.82 (0.004)
0.2–0.3	0.86 (0.003)
0.3–0.4	0.88 (0.003)
0.4–0.5	0.83 (0.003)

^1^ Minor allele frequency

^a^ Values are the average across 10 random subsets of animals

^b^ The values in parentheses are standard errors

#### Relationship between the reference and validation population

The average of the top five genomic relationships of a given animal in the validation population with all animals in the reference population Ref_22_ was 0.14, 0.13, and 0.11 for G0, G1, and G2, respectively. With Ref_62_, these averages were 0.21, 0.16, and 0.13 for G0, G1, and G2, respectively. Although the average top five relationships decreased across generations, average accuracies did not follow this declining pattern with more distant validation generations. From G0 to G1, the average imputation accuracies across all MAF classes reduced by 0.04 for both Ref_22_ and Ref_62_. From G1 to G2, the average accuracies increased by 0.05 for Ref_22_, and by 0.02 for Ref_62_ (Table 2). Also, only small differences in imputation accuracy were observed between animals that had only their sire, only their maternal grandsire, or both these ancestors in the reference. Imputation accuracy in the 48K to 60K scenario for these groups of animals was always within 0.02 of the accuracy observed across the whole validation population (Table 4). Also, in the 3K to 60K scenario, the imputation accuracies were nearly the same for these three groups (Table 4).

**Table 4:** Animal-specific imputation accuracy (r_corrected_) of G0 for three groups depending on their direct ancestors in the reference population Ref_62_

MAF^1^ class	GR_S^2^ (*N* ^3^ = 34)	GR_MGS^4^ (*N* = 23)	GR_SMGS^5^ (*N* = 310)
0.008–0.1	0.80	0.79	0.80
0.1–0.2	0.89	0.90	0.89
0.2–0.3	0.90	0.92	0.91
0.3–0.4	0.93	0.93	0.92
0.4–0.5	0.91	0.91	0.89
3K to 60K scenario	0.62	0.62	0.64

^1^ Minor allele frequency

^2^ Animals who had just their sire (S) in the reference population

^3^ N is the number of animals

^4^ Animals who had just their maternal grand sire (MGS) in the reference population

^5^ Animals who had both their sire and maternal grandsire (SMGS) in the reference population

#### Minor Allele Frequency (MAF)

Imputation accuracies were lower when MAF of the masked SNPs was lower. SNPs with low MAF were more difficult to impute correctly (Table 2) and exhibited more variation in their accuracy of imputation (Fig. 1). The difference in imputation accuracy for low and higher MAF SNPs was smaller with the larger reference, showing that even if imputation accuracy is already moderate for higher MAF SNPs, the accuracy for low MAF SNPs can still be improved by increasing the reference size. When SNPs were masked and evaluated based on their MAF in the validation population, instead of in the reference population, the average imputation accuracies across MAF classes were slightly reduced, by 0.01 on average (Additional file 2: Table S3). Compared with the scenario where SNPs were masked based on their MAF in the reference population (Table 2), an increase in the accuracy was observed when SNPs were masked independent of their MAF. Average accuracies (average across MAF classes) were higher by 0.08 and 0.04 for Ref_22_ and Ref_62_, respectively (Table 5). Again, the benefit was larger for SNPs with lower MAF and within the smaller reference population (Ref_22_).

**Table 5:** Animal-specific imputation accuracy (r_corrected_) with SNPs masked across the different MAF classes when G0 validation population was used for imputation

MAF^1^ class	Ref_22_	Ref_62_
0.008–0.1	0.80 (193)^a^	0.87 (186)
0.1–0.2	0.91 (178)	0.94 (177)
0.2–0.3	0.92 (181)	0.95 (180)
0.3–0.4	0.93 (186)	0.96 (189)
0.4–0.5	0.93 (184)	0.96 (194)

^1^ Minor allele frequency

^a^ The numbers in the parentheses are the number of masked SNPs

### Parameter to measure imputation accuracy

**Fig. 2:**
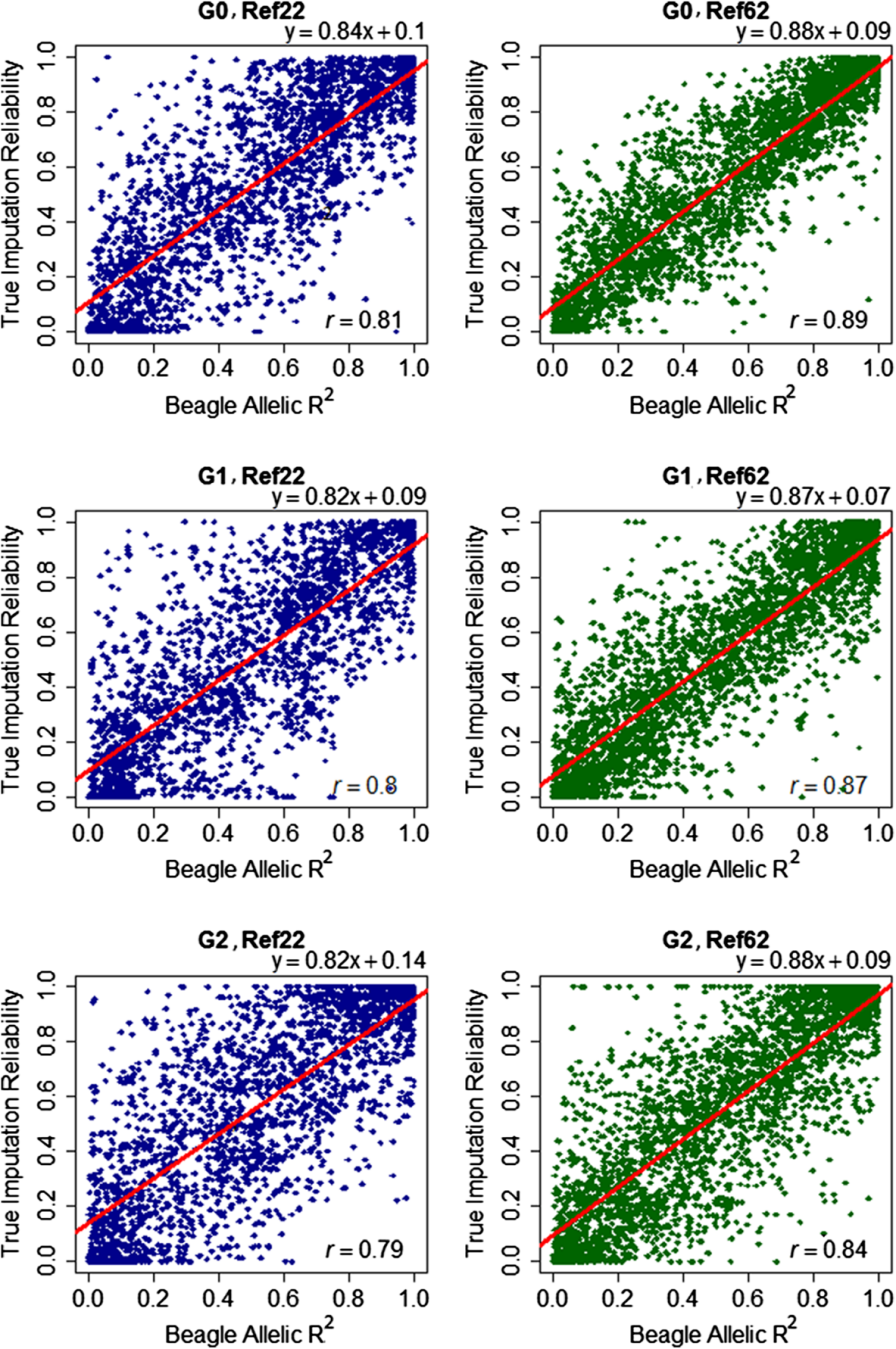
Correlation between true imputation reliability and allelic R^2^ measure from Beagle. True imputation reliability is plotted against the allelic R^2^ when 96 % of SNPs were masked (3K to 60K scenario) in G0, G1 and G2. The red line is the regression line

Our main measure of accuracy, r_corrected_, can only be measured when masking data in an experimental setting, which means it cannot be computed for common imputation tasks where the true genotypes are unknown. The Beagle software, however, estimates the “allelic R^2^” value, based on the posterior probability of the most likely genotype (see Methods). The allelic R^2^ predicts the reliability of imputed genotypes, and we compared it with the mean imputation reliabilities that were obtained as the squared correlation between true and imputed genotypes for each SNP (Table 6). Overall, the allelic R^2^ slightly overestimated the empirical imputation reliabilities across generations and reference populations. Average values of allelic R^2^ (average across generations) ranged from 0.64 to 0.82 for Ref_22_ and from 0.75 to 0.90 for Ref_62_ compared with empirical imputation reliabilities ranging from 0.59 to 0.81 and from 0.68 to 0.85, respectively (Table 6). For SNPs with higher MAF, the two measures were more similar than for SNPs with low MAF. For instance, the difference between the two measures was as much as 0.05 for low MAF (< 0.1) and only 0.02 for high MAF (0.4-0.5), when Ref_22_ was used for imputation. In general, the correlation between the two measures was moderate to high depending on the SNP density of the validation population. In the 48K to 60K scenario, the correlation between the allelic R^2^ and the imputation reliability was on average (across different MAF classes) 0.70, 0.69 and 0.58 in G0, G1, and G2, respectively, using Ref_22_. By increasing the reference size (Ref_62_), the correlation increased by 0.06, 0.05, and 0.09 in G0, G1, and G2, respectively (Table 7). Correlations between the allelic R^2^ and the imputation reliability were higher in the 3K to 60K scenario, compared with the 48K to 60K scenario, with increases of 0.11, 0.11 and 0.21 in G0, G1, and G2 using Ref_22_, and by 0.13, 0.13, and 0.17 in G0, G1, and G2 using Ref_62_, respectively (Fig. 2).

**Table 6:** Average allelic R^2^ measure from Beagle and true imputation reliability on GGA1 for different MAF classes and different reference sizes (48K to 60K scenario)

	Ref_22_		Ref_62_	
MAF^1^ class	Reliability^a^	Allelic R^2^	Reliability	Allelic R^2^
0.008–0.1	0.59	0.64	0.68	0.75
0.1–0.2	0.73	0.77	0.79	0.85
0.2–0.3	0.78	0.80	0.83	0.88
0.3–0.4	0.81	0.82	0.85	0.90
0.4–0.5	0.79	0.81	0.83	0.87

^1^ Minor allele frequency

^a^Reliability is the square of imputation accuracy per SNP across individuals (SNP-specific imputation accuracy), i.e. the imputation accuracy per SNP was squared and were then summed across individuals. Note that the values in this table are average across the three generations (G0, G1 and G2)

**Table 7:** Correlation between allelic R^2^ measure from Beagle and true imputation reliability on GGA1 for different MAF classes and different reference sizes in G0, G1 and G2 (48K to 60K scenario)

	Ref_22_			Ref_62_		
MAF^1^class	G0^2^	G1^3^	G2^4^	G0	G1	G2
0.008–0.1	0.70	0.60	0.45	0.67	0.71	0.51
0.1–0.2	0.67	0.73	0.52	0.72	0.72	0.63
0.2–0.3	0.75	0.72	0.64	0.74	0.73	0.71
0.3–0.4	0.64	0.69	0.60	0.79	0.76	0.68
0.4–0.5	0.74	0.72	0.71	0.85	0.81	0.82

^1^ Minor allele frequency

^2^ First generation of genomic selection experiment

^3^ Offspring of G0

^4^ Offspring of G1

### Size of the chromosome

Imputation accuracies were obtained for GGA8 to investigate whether the imputation results for GGA1 were representative for other chromosomes. For GGA8, a similar pattern of accuracies was observed across generations, and across MAF classes. Average imputation accuracies across MAF classes were slightly smaller, by ~ 0.01, for SNPs on GGA8 across all generations (Additional file 3: Table S4).

## Discussion

Several SNP chips with different densities (42K, 60K and 600K) have been developed for chicken and additional chips may be developed in the near future. In this study, we mimicked the imputation of genotypes between two different SNP chips with similar densities by imputing from 48K to 60K. We were specifically interested in imputation of low MAF SNPs when imputing towards one of the chips, because SNPs with low frequency may play an important role in complex traits and may have larger effects than the common SNPs in a population [42]. In addition, the accuracy of imputation of the 60K genotypes from a very low density SNP panel (3K) was assessed. In both scenarios (3K to 60K and 48K to 60K), imputation was performed using a small reference population of white layer chickens. The reference animals were carefully selected to include recent ancestors (sires and MGS of G0) or a subset thereof, chosen based on the proportion of their contributions to the validation animals. The results indicate that genotype imputation based on a small number of carefully selected reference animals resulted in low imputation accuracy for the 3K to 60K scenario (between 0.46 to 0.50 for Ref_22_ and from 0.58 to 0.63 for Ref_62_) and in moderate imputation accuracy for the 48K to 60K scenario (between 0.60 to 0.89 for Ref_22_ and from 0.73 to 0.93 for Ref_62_).

Several studies have reported reasonable accuracies of imputation of SNP genotypes between different SNP chips in cattle [3, 26, 43]. For instance, Khatkar et al. [26] found error rates of 2.75 % and 0.76 % when imputing from 25K to 50K and from 35K to 50K, respectively. Druet et al. [3] found an error rate of 1 % when imputing from 50K to 60K. Also, in beef cattle, imputation from the public BovineSNP50K BeadChip to a proprietary 50K panel yielded imputation accuracies (allelic R^2^) in the range of 0.94 to 0.98 [43]. In all these studies, the reference populations were much larger than the reference population used in our study.

Past studies showed that imputation accuracy depends on the size of reference population, the level of relationship between the reference and validation populations, and MAF of the SNP being imputed [18, 19, 21, 44]. In the current study, imputation accuracy depended on the size of reference population and the MAF of the SNP being imputed, but did not depend on the level of the relationship between the reference and validation populations. With Ref_22_, only little variation in the top five relationships was observed, while variation in the top five relationships was larger when Ref_62_ was used as reference population. However, with both Ref_22_ and Ref_62_, the imputation accuracy did not follow the pattern of variation in relationships. We found that the size of reference population was more important for obtaining higher accuracy when the validation population was genotyped at lower density (3K). With a higher SNP density in the validation populations (48K), the impact of reference size on imputation accuracy was less, showing that the factors influencing the imputation accuracy interact with each other.

When the size of the reference population was small, the pedigree-free imputation method implemented in Beagle yielded low to moderate imputation accuracy. Badke et al. [45] obtained high imputation accuracy with two small reference populations consisting of 16 or 64 Yorkshire pigs with phased genotype data. Imputing the genotypes of a validation population (*n* = 200) resulted in accuracies of 0.90 and 0.95 using Beagle’s default parameters [45]. In their data, the reference included both parents of all the validation animals, which probably has a beneficial effect on the imputation accuracy. This benefit could not be tested in our data, because female parents were not genotyped. In addition to having both parents in the reference, the use of a phased reference population is a factor that is expected to increase the imputation accuracy compared with our results [33].

### Factors affecting the imputation accuracy

#### Size of reference population

Increasing the size of the reference population decreases the probability to miss a haplotype in the reference population [46] and increases the probability that multiple copies of alleles are present for making the correct haplotypes [47]. As expected, the accuracy of imputation increased with the size of reference population for both 3K to 60K and 48K to 60K scenarios, which is in agreement with other studies [19, 20, 27]. For example, in G0, the increase in average imputation accuracies (average across MAF classes) was 0.07 (from 0.82 to 0.89). With the 3K to 60K scenario, the average increase in imputation accuracy was larger (e.g. from 0.50 to 0.63 for G0; Table 1) from increasing the reference population from 22 to 62, indicating that when a lower density SNP panel is used for imputation, a larger number of individuals in reference population can, at least in part, make up for the reduced imputation accuracy. Beagle has been extensively applied to impute missing genotypes in human and animal genetics, and imputation accuracy with small reference populations has been reported to be moderate to high. Hayes et al. [19] obtained an imputation accuracy of ~ 0.8 when the reference population consisted of only 25 or 40 Border Leicester sheep. Vereijken et al. [39] used 57 brown layers to impute the missing genotypes of 249 animals and obtained a SNP-specific imputation accuracy in the range of 0.75 to 0.9 (average across different chromosomes) with different panel densities. While moderate imputation accuracies were observed in these studies, it has also been shown that with a very small reference population, the application of an appropriate imputation method is crucial [20]. With a small reference population, Beagle did not result in the highest imputation accuracies in a study on dairy cattle data [20].

Accuracies were higher with our Ref_22_ compared with the randomly selected reference populations, Ref_22rand_. There was no improvement in accuracy for the classes with MAF > 0.10, except for a small improvement of 0.03 for MAF class 0.4-0.5. The largest increase of 0.07 was found for the lowest MAF class (MAF < 0.10), indicating that including the most common sires as a reference population will mostly benefit the imputation of the most difficult class of SNPs, those with lower MAF. Pausch et al. [20] showed, in Fleckvieh cattle, that pre-selecting key animals was slightly beneficial for subsequent genotype imputation.

The required size of the reference population to achieve high imputation accuracy differs across populations and has been suggested to depend mainly on the effective population size, *N*_*e*_ [48], which is relatively low for this population (52). In populations with small *N*_*e*_, genotype imputation based on a small number of carefully selected reference animals was shown to yield a reasonable accuracy [49].

#### Relationship between the reference and validation population

Several studies have shown that the relationship between the reference and validation populations influences the imputation accuracy in sheep [19], maize [21], beef cattle [44] and dairy cattle [26–28]. All these studies reported that the accuracy of imputation was greatest for individuals with the highest average genetic relationship to the reference population, which was attributed to them sharing more and longer haplotypes with the reference. Ventura et al. [44] reported that with removal of the 37 close relatives from the reference population of 313 Angus cattle, the imputation accuracy decreased by 2.3 % using Beagle. The reason given for this decrease in accuracy was that close relatives introduce conserved long haplotypes in the reference population, favouring an effective haplotype search in the imputation process [44]. In our dataset, however, only small differences in imputation accuracy were observed when animals had only their sire, only their maternal grandsire, or both these ancestors in the reference. One possible reason that the imputation accuracies are so similar among these three groups might be the small number of individuals in each of these groups which makes it hard to compare the imputation accuracies.

**Fig. 3:**
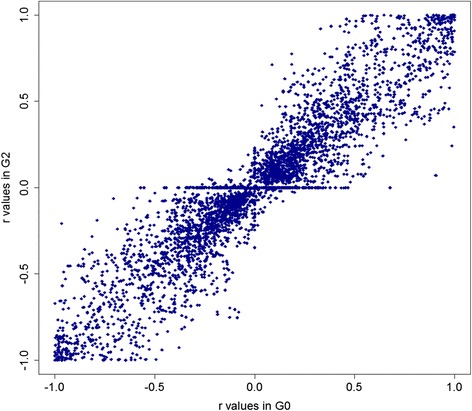
Concordance of LD in G0 and G2. LD within each generation was measured as r (correlation) [51] between neighbouring SNPs

Instead of the average relationship with the whole reference population, we compared imputation accuracy across the three generations with the average of the top five relationships. It has been shown that this measure correlates better with the accuracy of genomic prediction compared with the mean relationship [50]. With Ref_62_, the top five relationships decreased from 0.21 in G0 to 0.16 in G1, and 0.13 in G2. The average imputation accuracies (average across MAF classes) showed only a small reduction between G0 and G1, from 0.82 to 0.78 for Ref_22_ and from 0.89 to 0.85 for Ref_62_. From G1 to G2, the average accuracies increased slightly, despite the reduction in the top five relationships. The persistence of imputation accuracy in later generations is desirable, and may be a feature of small populations that are closed such that most common sires can be put in the reference. With a pedigree-based imputation method, the distance to the reference population might have had more impact on the imputation accuracy, because pedigree-based methods were found to be more dependent on having close relatives in the reference population than pedigree-free imputation methods [18]. Another factor that can explain the persistence of accuracies with increasing distance to the reference population is the high persistence of LD across generations (Fig. 3). Animals that are several generations apart will still share haplotypes, at least over short distances, and population level LD will hence only change slowly. For the calculation of LD measured as *r* [51], phased and imputed SNP data were used as described in [52]. Correlation (concordance) between values of *r* estimated in G0 or G2 was 0.93 (Fig. 3). For pedigree-free imputation algorithms such as Beagle, the LD pattern in the data is the only information that is explicitly used, although it has been shown that the LD-based imputation methods use the relationship information indirectly [26]. With higher LD, the algorithm can better identify the haplotypes, which is easier with 60K data in the validation population, compared with 1K and 3K in previously reported studies [19, 39]. In addition, it was argued that as the density of the validation panel increases, the effects of genetic relatedness will be less important, because at higher density shorter haplotypes can be imputed correctly, which makes it possible for haplotypes from more distantly related individuals to be imputed correctly [21].

Our reason for imputing to higher density is to improve accuracies in genomic prediction scenarios. High imputation accuracy is required in later generations to achieve accurate prediction of genomic breeding values in those generations. Wolc et al. [9] did not apply imputation, but they did find the accuracy of genomic estimated breeding values (GEBV) for brown layers to be persistent between generations two to five after the training data using real genotypes (42K SNP chip data). This result was obtained with real genotypes in all generations but it indicates that if imputation accuracy is high, prediction accuracy can be expected to also be persistent in later generations [9].

#### Minor Allele Frequency (MAF)

It has been suggested that SNPs with low frequency may play an important role in complex traits, and may have larger effects than the common SNPs in a population [42]. Hence, we were specifically interested in the accuracy of imputed genotypes for SNPs with low MAF. Accuracies of imputation were lower when MAF of the masked SNPs was lower, which may be due to a lower degree of LD with the 60K SNPs (selected for higher MAF), or due to a more challenging haplotype reconstruction when few haplotypes carry the minor allele. Inclusion of very rare SNPs may interfere with phasing, resulting in less accurately constructed haplotypes and ultimately leading to inferior imputation quality [53]. The decline in the imputation accuracy for lower MAF was smaller when the reference size was larger showing that the imputation accuracy probably depends more strongly on the number of copies of the minor allele in the reference population than the MAF itself.

The lower imputation accuracy when MAF was low is in agreement with other studies that used chip data [18–23] and sequence data [24] in different species. However, various measures of the imputation accuracy were used in those studies, hampering a quantitative comparison. In this study, where we used the correlation coefficient corrected for gene content, a small decrease in imputation accuracy was observed with MAF < 0.1 compared with higher MAF SNPs. In another analysis with the same data, we observed a greater decrease in imputation accuracy for MAF < 0.05 [54]. Lin et al. [23] showed that the decline in imputation accuracy already started with MAF < 0.15 in human data. Hickey et al. [21] and Hayes et al. [19] also reported the decline in imputation accuracy for MAF < 0.1 in maize and sheep populations. Interestingly, the selection of the most common sires appears to especially benefit imputation accuracy of low MAF SNPs.

Small differences in imputation accuracies were observed when SNPs were masked based on their MAF in the validation population, instead of in the reference population. Since the fraction of the SNPs that was monomorphic in Ref_22_ and Ref_62_, but polymorphic in the validation population (G0) was relatively low (3.86 % in Ref_22_ and 1.07 % in Ref_62_), little difference in imputation accuracies was expected by masking MAF from the validation populations. When SNPs were masked independent of their MAF, imputation accuracy was larger for SNPs with lower MAF and within the smaller reference population (Ref_22_) (Table 5), indicating that SNPs with low MAF can be imputed more accurately when SNPs with different ranges of MAF were used to impute them. This suggests that a genotyping panel to be used for imputing to higher densities should not contain SNPs with intermediate frequencies, as has been done for the currently available SNP chips.

### Comparison of true reliability and allelic R^2^ from Beagle

The correlation between the allelic R^2^ reported by Beagle and the imputation reliability calculated in this study was moderate to high, (Fig. 2 (3K to 60K scenario) and Table 7 (48K to 60K scenario)). The correlations were higher when the reference size was larger and the MAF was higher, which is in agreement with [24]. Further, the correlations tended to be higher when the validation density was lower (3K to 60K). For the 3K to 60K scenario, the regression of imputation reliability on allelic R^2^ was close to 1 (low bias), ranging from 0.82 to 0.88 in different scenarios (Fig. 2), which allows us to predict the reliability when the true genotypes of missing SNPs are unknown. Hence, with a very low-density reference panel (e.g. 3K) allelic R^2^ may be used as a measure of accuracy when validation using masked data is not possible. For instance, imputation of all genotyped animals in a validation population using a small number of sequenced animals does not allow comparison with the true genotypes of the non-sequenced animals, and the reference population is typically too small to allow cross-validation.

### Size of the chromosome

In this study, imputation accuracy was not very different between chromosomes of different size, which is in agreement with [39]. However, a study in Angus cattle [55] showed that there is a positive association between the chromosome size and the imputation accuracy. The reported differences between the imputation accuracies on large and small chromosomes were, however, not large (less than 0.02 using Beagle) [55]. The reason for a slightly lower accuracy on smaller chromosomes would be the reduced accuracy at the beginning and end of the chromosome which would have a relatively larger effect for small chromosomes. In another study in cattle, it was shown that the number of SNPs per centi-Morgan influenced imputation error rate more than the chromosome size [30].

## Conclusions

In a scenario to mimic the imputation of genotypes between different SNP chips of similar densities, we found that moderate levels of imputation accuracy can be achieved even with a very small number of animals in the reference population. Selecting animals for the reference population from the most common sires, rather than selecting random animals for the reference population, considerably improved imputation accuracy for SNPs with low MAF, and slightly for SNPs with the highest MAF. Accuracy could be further increased by adding animals to the reference population particularly when the validation population was genotyped for a low-density panel (3K) or the SNPs targeted for imputation had low MAF. The allelic R^2^ estimated by Beagle gave a good indication of imputation reliability when the density of validation panel was very low (3K) and the MAF of the SNP and the size of the reference population were not extremely small.

## Methods

### Data

The study was performed with data from a commercial white layer line of chicken. Animals that were genotyped with the Illumina Infinium iSelect Beadchip (60K chip) (Illumina Inc., San Diego, CA, USA) [4] came from four generations of training data, preceding the three generations of selection candidates (G0, G1, and G2) which were selected by genomic best linear unbiased prediction (GBLUP) method. Total number of genotyped animals was 2140. More details about the structure of data are in [54].

### Quality control

Data from 8623 SNPs on chromosome 1 (GGA1) and 1700 SNPs on chromosome 8 (GGA8) were used to assess imputation accuracy on two chromosomes of very different size. SNPs were removed if they had a MAF < 0.01, a call rate < 0.9, or > 10 % parent-progeny Mendelian inconsistencies. Animals were removed if their genotype call rate was < 0.9. After filtering, 4485 SNPs on GGA1, 824 SNPs on GGA8, and 2140 animals remained for further analyses.

### Selection of animals for the reference population

Of 2140 genotyped animals, 62 were sires and/or maternal grand sires (MGS) of animals in G0. The actual number of sires and maternal grandsires of G0 was 67, but 5 of them had no DNA sample available. Of these 62 sires and maternal grandsires, 22 most common sires were chosen as the reference population (Ref_22_). These 22 most common sires will be sequenced for further investigation of GS with (imputed) whole-genome sequence data. Ref_22_ was chosen based on their “proportion of genetic diversity” [56] in order to capture the greatest possible proportion of genetic variation in the target population. Capturing a large part of the genetic variation by selecting the most common sires should provide a high accuracy of genotype imputation. The details of the method are described in the next section. For this study, imputation was performed using 60K genotype data on GGA1 and GGA8. The results obtained from 22 reference animals were compared with the results obtained with 62 reference animals.

### Proportion of genetic diversity

The genomic relationship matrix from SNPs (**G** matrix) [57] was obtained for 2140 genotyped animals. The proportion of diversity was calculated as: P_n_ = G_n_^−1^ c_n_, where **G**_**n**_ was a subset of the genomic relationship matrix (*n* = 62 genotyped sires and maternal grandsires), **c**_**n**_ was a vector with the average genomic relationship of the **n** sires and maternal grandsires with the target population, and **P**_**n**_ was a vector of the proportion of the genetic diversity captured by the **n** sires and maternal grandsires.

### Imputation scenarios

#### Imputation from 3K to 60K

In the “3K to 60K” scenario, imputation from a very low-density SNP panel (i.e. a 3K panel) to a medium density SNP panel (60K) was tested by masking ~ 96 % of 60K SNPs in a structured way (virtually designed and evenly spaced) across the genome. The same reference and validation populations were used as above.

#### Imputation from 48K to 60K

The imputation accuracy from the “48K to 60K” scenario was compared with those from 3K to 60K scenario to investigate the impact of SNP density in the reference on imputation accuracy. Moreover, imputation from 48K to 60K mimics the imputation of genotypes between two different SNP chips with similar densities. In five different classes of MAF (see next section), each containing approximately 20 % of all the SNPs, genotypes were set to missing in the validation population, creating five panels of 48K SNPs.

### Factors affecting the imputation accuracy

#### Size of reference population

Imputation accuracy was assessed when using the 62 sires and maternal grandsires (Ref_62_), or Ref_22_ as the reference population. In an additional analysis, with validation population G0, 22 animals were randomly selected as reference population from the training population (that consisted of the four generations before G0) which included the 62 common sires. The random selection of reference animals and subsequent genotype imputation and validation was repeated ten times (Ref_22rand_).

#### Relationship between the reference and validation population

The three validation populations consisted of the animals in consecutive generations G0, G1, and G2. The number of animals in G0, G1 and G2 were 367, 395 and 148, respectively. Comparison of imputation accuracies in G0, G1 and G2 will give an insight on the effect of distance to the reference population on imputation accuracy. Further, to assess the impact of an animal’s relationship to the reference population on imputation accuracy, accuracies were determined within each generation and compared with a measure of genomic relatedness which was the average of the top five relationships [50] with animals in the reference. Additionally, imputation accuracy was also computed for three groups of G0 animals, separated by the type of direct ancestors they had in the reference population Ref_62_: (1) animals who had just their sire (GR_S, *n* = 34), (2) just their maternal grand sire (GR_MGS, *n* = 23), or (3) both their sire and maternal grandsire (GR_SMGS, *n* = 310) in the reference population.

#### Minor Allele Frequency (MAF)

The relationship between MAF of SNPs to be imputed and the imputation accuracy was investigated by masking SNPs in five different classes of MAF ranging from 0.008 to 0.5: [0.008-0.1], [0.1-0.2], [0.2-0.3], [0.3-0.4], and [0.4-0.5] (Additional file 4: Table S1). Imputation was done separately for all combinations of the two reference populations (Ref_22_ and Ref_62_), the three validation populations (G0, G1, and G2), and the five MAF classes. To investigate the impact of choosing SNPs to mask on imputation accuracy, some scenarios were repeated with: first, SNPs being masked based on their MAF in the G0 validation population instead of the reference, and second, SNPs being masked independent of their MAF class, i.e. SNPs from all different MAF ranges were masked and imputed in one analysis. Imputation accuracy was then computed within different MAF classes. In all these scenarios, approximately 20 % of all the SNPs from the 60K panel were set to missing in the validation population. As mentioned earlier, these scenarios were therefore identified as 48K to 60K scenarios.

### Imputation methods

Masked SNPs were imputed using Beagle version 3.3.2 [33]. Beagle uses a localized haplotype cluster model to cluster haplotypes at each marker and then defines a hidden Markov model (HMM) to find the most likely haplotype pairs based on the individual’s known genotypes. Beagle predicts the most likely genotype at missing SNPs from defined haplotype pairs [33]. In our previous study [54], we showed that the accuracy of imputation was very low in a preliminary analysis that applied the default parameters. We therefore tested several parameter settings of Beagle for the current analyses. Most importantly, Beagle was run for 50 iterations of the phasing algorithm rather than the default number of 10 iterations. Changing other parameters such as increasing the number of samples (number of haplotype pairs to sample for each individual during each iteration of the phasing algorithm) and number of imputations (average the posterior probabilities over multiple imputations) was also tested. However, we found no increase in imputation accuracy when these parameters were changed and default settings were therefore applied [54].

### Measure of imputation accuracy

Animal-specific imputation accuracy (r_corrected_), computed as the correlation between the true genotypes (coded as 0, 1, or 2 minus the mean gene content) and the imputed genotype (the most likely genotype minus the mean gene content) as suggested by Mulder et al. [28], was used as the measure of imputation accuracy. Mean gene content was computed per SNP as the mean of the genotypes represented as 0, 1, and 2, and was based on genotyped reference animals in each scenario. The reason for correction (subtracting the mean gene content from true and imputed genotypes) is that different SNPs have different MAF and therefore SNPs have distributions with different means. By correcting for the gene content, it is assumed that the correlated variables are bivariate normally distributed. Besides calculating animal-specific imputation accuracy for each individual, the imputation accuracy was also computed per SNP across individuals (SNP-specific imputation accuracy). SNP-specific imputation accuracy was computed as the correlation between the true and imputed genotypes (the most likely genotype) for each masked SNP coded as 0, 1 and 2 for genotypes A_1_A_1_, A_1_A_2_, and A_2_A_2_, respectively. We then compared the square of SNP-specific imputation accuracy (“true” imputation reliability) with allelic R^2^ generated by Beagle. Allelic R^2^ is the squared correlation between the allele dosage of the most likely imputed genotype and the allele dosage of the true genotype. The estimated A_2_-allele dosage was obtained from the imputed posterior genotype probabilities as: 0 * P(A_1_A_1_) + 1 * P(A_1_A_2_) + 2 * P(A_2_A_2_) [33]. The results of r_corrected_ were given and discussed throughout this paper as the main measure of imputation accuracy for different scenarios. Allelic R^2^ was compared with true imputation reliability in a separate section (see Discussion).

### Calculation of effective population size (*N*_*e*_)

*N*_*e*_ was estimated from the observed LD values (*r*^2^) between SNPs. The *r*^2^ was related to *N*_*e*_ based on Sved’s equation [58]:

$$ {r}^2=\frac{1}{1+4{N}_ec} $$

The genetic distance between SNPs (*c*, in Morgan units) was obtained by converting the physical distances (in base-pairs) to genetic distances (in Morgan) using the recombination rate values as reported by International Chicken Genome Sequencing Consortium (ICGSC) [59]. This estimate of *N*_*e*_ has been obtained under the assumption of constant population size [58].

### Ethics statement

Blood samples were collected as part of routine data and sample collection in a commercial breeding program. According to the local legislation, it was not needed to have permission from the ethics committee.

## Additional files

Additional file 1: Table S2. Proportion of diversity for 62 sires and maternal grand sires (MGS) of G0. Additional file 2: Table S3. Animal-specific imputation accuracy (r_corrected_) for SNPs classified by MAF in validation population Additional file 3: Table S4. Animal-specific imputation accuracy (r_corrected_) on GGA8 for different MAF classes and different reference sizes in G0, G1 and G2. Additional file 4: Table S1. Total number of SNPs masked for different MAF classes in 48K to 60K scenario.

## Abbreviations

GS: Genomic selection
GWAS: Genome-wide association study
MAF: Minor allele frequency
SNP: Single nucleotide polymorphism
LD: Linkage disequilibrium
QTL: Quantitative trait loci
*N*_*e*_: Effective population size
GBLUP: Genomic best linear unbiased prediction
MGS: Maternal grand sire
G: Genomic relationship matrix
HMM: Hidden Markov model
GEBV: Genomic estimated breeding values
ICGSC: International chicken genome sequencing consortium
