# Supplementary material for: Accuracy of imputation using the most common sires as reference population in layer chickens
Source: BMC Genet. 2015 Aug 18;16:101. doi: 10.1186/s12863-015-0253-5 (PMC4539854; doi:10.1186/s12863-015-0253-5)
Supplement: Additional file 1: Table S2. — Proportion of diversity for 62 sires and maternal grand sires (MGS) of G0. [file 12863_2015_253_MOESM1_ESM.docx]

| **Class** | **MAF^1^** | **Number of masked SNPs**^2^ **(Ref_22_)** | **Total number of SNPs** | **Percentage of Masked SNPs** | **Number of masked SNPs (Ref_62_)** | **Total number of SNPs** | **Percentage of Masked SNPs** |
| --- | --- | --- | --- | --- | --- | --- | --- |
| 1 | 0.008-0.1 | 772 | 4485 | 0.17 | 837 | 4485 | 0.19 |
| 2 | 0.1-0.2 | 887 | 4485 | 0.20 | 885 | 4485 | 0.20 |
| 3 | 0.2-0.3 | 1081 | 4485 | 0.24 | 990 | 4485 | 0.22 |
| 4 | 0.3-0.4 | 835 | 4485 | 0.19 | 850 | 4485 | 0.19 |
| 5 | 0.4-0.5 | 733 | 4485 | 0.17 | 873 | 4485 | 0.19 |

^1^ Minor allele frequency.

^2^ Single nucleotide polymorphisms.
