# Supplementary material for: Accuracy of imputation using the most common sires as reference population in layer chickens
Source: BMC Genet. 2015 Aug 18;16:101. doi: 10.1186/s12863-015-0253-5 (PMC4539854; doi:10.1186/s12863-015-0253-5)
Supplement: Additional file 2: Table S3. — Animal-specific imputation accuracy (rcorrected) for SNPs classified by MAF in validation population [file 12863_2015_253_MOESM2_ESM.docx]

| **Animal** | **Proportion of diversity** | **Animal** | **Proportion of diversity** |
| --- | --- | --- | --- |
| 1 | 0.0277 | 32 | 0.0116 |
| 2 | 0.0267 | 33 | 0.0115 |
| 3 | 0.0242 | 34 | 0.0113 |
| 4 | 0.0214 | 35 | 0.0112 |
| 5 | 0.0211 | 36 | 0.0110 |
| 6 | 0.0199 | 37 | 0.0107 |
| 7 | 0.0196 | 38 | 0.0104 |
| 8 | 0.0187 | 39 | 0.0101 |
| 9 | 0.0186 | 40 | 0.0099 |
| 10 | 0.0186 | 41 | 0.0097 |
| 11 | 0.0173 | 42 | 0.0095 |
| 12 | 0.0165 | 43 | 0.0095 |
| 13 | 0.0165 | 44 | 0.0093 |
| 14 | 0.0152 | 45 | 0.0088 |
| 15 | 0.0151 | 46 | 0.0084 |
| 16 | 0.0149 | 47 | 0.0082 |
| 17 | 0.0149 | 48 | 0.0081 |
| 18 | 0.0148 | 49 | 0.0080 |
| 19 | 0.0145 | 50 | 0.0079 |
| 20 | 0.0145 | 51 | 0.0077 |
| 21 | 0.0141 | 52 | 0.0077 |
| 22 | 0.0141 | 53 | 0.0076 |
| 23 | 0.0135 | 54 | 0.0065 |
| 24 | 0.0133 | 55 | 0.0061 |
| 25 | 0.0133 | 56 | 0.0061 |
| 26 | 0.0121 | 57 | 0.0053 |
| 27 | 0.0120 | 58 | 0.0039 |
| 28 | 0.0119 | 59 | 0.0029 |
| 29 | 0.0118 | 60 | 0.0027 |
| 30 | 0.0118 | 61 | 0.0025 |
| 31 | 0.0116 | 62 | 0.0018 |
