# Supplementary material for: Accuracy of imputation using the most common sires as reference population in layer chickens
Source: BMC Genet. 2015 Aug 18;16:101. doi: 10.1186/s12863-015-0253-5 (PMC4539854; doi:10.1186/s12863-015-0253-5)
Supplement: Additional file 3: Table S4. — Animal-specific imputation accuracy (rcorrected) on GGA8 for different MAF classes and different reference sizes in G0, G1 and G2. [file 12863_2015_253_MOESM3_ESM.docx]

| **Class** | **MAF^1^** | **Ref_22_** | **Ref_62_** |
| --- | --- | --- | --- |
| 1 | 0.008-0.1 | 0.67 | 0.82 |
| 2 | 0.1-0.2 | 0.81 | 0.88 |
| 3 | 0.2-0.3 | 0.84 | 0.91 |
| 4 | 0.3-0.4 | 0.85 | 0.91 |
| 5 | 0.4-0.5 | 0.83 | 0.89 |

^1^ Minor allele frequency. SNPs were masked and evaluated based on their MAF in the validation population, instead of in the reference population.
