# Supplementary material for: Accuracy of imputation using the most common sires as reference population in layer chickens
Source: BMC Genet. 2015 Aug 18;16:101. doi: 10.1186/s12863-015-0253-5 (PMC4539854; doi:10.1186/s12863-015-0253-5)
Supplement: Additional file 4: Table S1. — Total number of SNPs masked for different MAF classes in 48K to 60K scenario. [file 12863_2015_253_MOESM4_ESM.docx]

|  |  | **Ref_22_** | | | **Ref_62_** | | |
| --- | --- | --- | --- | --- | --- | --- | --- |
| **Class** | **MAF^1^** | **G0^2^** | **G1^3^** | **G2^4^** | **G0** | **G1** | **G2** |
| 1 | 0.008-0.1 | 0.59 | 0.51 | 0.62 | 0.74 | 0.80 | 0.69 |
| 2 | 0.1-0.2 | 0.86 | 0.86 | 0.83 | 0.87 | 0.87 | 0.88 |
| 3 | 0.2-0.3 | 0.83 | 0.82 | 0.85 | 0.90 | 0.89 | 0.90 |
| 4 | 0.3-0.4 | 0.87 | 0.87 | 0.90 | 0.91 | 0.86 | 0.90 |
| 5 | 0.4-0.5 | 0.90 | 0.88 | 0.92 | 0.88 | 0.87 | 0.91 |

^1^ Minor allele frequency.

^2^ First generation of genomic selection experiment.

^3^ Offspring of G0.

^4^ Offspring of G1.
